# A dose-finding Phase 2 study of single agent isatuximab (anti-CD38 mAb) in relapsed/refractory multiple myeloma

**DOI:** 10.1038/s41375-020-0857-2

**Published:** 2020-05-14

**Authors:** Joseph Mikhael, Joshua Richter, Ravi Vij, Craig Cole, Jeffrey Zonder, Jonathan L. Kaufman, William Bensinger, Meletios Dimopoulos, Nikoletta Lendvai, Parameswaran Hari, Enrique M. Ocio, Cristina Gasparetto, Shaji Kumar, Corina Oprea, Marielle Chiron, Claire Brillac, Eric Charpentier, Jesús San-Miguel, Thomas Martin

**Affiliations:** 1 Mayo Clinic, Scottsdale, AZ USA; 2 Hackensack University Medical Center, Hackensack, NJ USA; 3 Washington University School of Medicine, St. Louis, MO USA; 4 University of Michigan, Ann Arbor, MI USA; 5 Karmanos Cancer Institute, Detroit, MI USA; 6 Winship Cancer Institute of Emory University, Atlanta, GA USA; 7 Fred Hutchinson Cancer Research Center, Seattle, WA USA; 8 National and Kapodistrian University of Athens School of Medicine, Athens, Greece; 9 Memorial Sloan-Kettering Cancer Center, New York, NY USA; 10 Medical College of Wisconsin, Milwaukee, WI USA; 11 Hospital Universitario Marqués de Valdecilla (IDIVAL), Universidad de Cantabria, Santander, Spain; 12 Duke University Medical Center, Durham, NC USA; 13 Mayo Clinic, Rochester, MN USA; 14 Sanofi Oncology R&D, Vitry/Alfortville, France; 15 Sanofi Genzyme Oncology, Cambridge, MA USA; 16 Clinica Universidad de Navarra, CIMA, IDISNA, CIBERONC, Pamplona, Spain; 17 University of California at San Francisco, San Francisco, CA USA; 18 Present Address: Translational Genomics Research Institute, City of Hope Cancer Center, Phoenix, AZ USA; 19 Present Address: Mount Sinai Hospital, New York, NY USA; 20 Present Address: Michigan State University, East Lansing, MI USA; 21 Present Address: Janssen Pharmaceuticals, Titusville, NJ USA

**Keywords:** Myeloma, Myeloma, Cancer immunotherapy

## Abstract

A Phase 2 dose-finding study evaluated isatuximab, an anti-CD38 monoclonal antibody, in relapsed/refractory multiple myeloma (RRMM; NCT01084252). Patients with ≥3 prior lines or refractory to both immunomodulatory drugs and proteasome inhibitors (dual refractory) were randomized to isatuximab 3 mg/kg every 2 weeks (Q2W), 10 mg/kg Q2W(2 cycles)/Q4W, or 10 mg/kg Q2W. A fourth arm evaluated 20 mg/kg QW(1 cycle)/Q2W. Patients (*N* = 97) had a median (range) age of 62 years (38–85), 5 (2–14) prior therapy lines, and 85% were double refractory. The overall response rate (ORR) was 4.3, 20.0, 29.2, and 24.0% with isatuximab 3 mg/kg Q2W, 10 mg/kg Q2W/Q4W, 10 mg/kg Q2W, and 20 mg/kg QW/Q2W, respectively. At doses ≥10 mg/kg, median progression-free survival and overall survival were 4.6 and 18.7 months, respectively, and the ORR was 40.9% (9/22) in patients with high-risk cytogenetics. CD38 receptor density was similar in responders and non-responders. The most common non-hematologic adverse events (typically grade ≤2) were nausea (34.0%), fatigue (32.0%), and upper respiratory tract infections (28.9%). Infusion reactions (typically with first infusion and grade ≤2) occurred in 51.5% of patients. In conclusion, isatuximab is active and generally well tolerated in heavily pretreated RRMM, with greatest efficacy at doses ≥10 mg/kg.

## Introduction

The introduction of proteasome inhibitors (PIs) and immunomodulatory agents (IMiDs) has improved clinical outcomes in patients with multiple myeloma (MM) [1], and 5-year survival rates have increased steadily to reach 52% for the 2009–2015 period [2]. However, survival declines with successive lines of therapy [3], and only a third of patients diagnosed with MM are expected to survive beyond 10 years [4]. Patients with MM that is dual refractory to IMiDs (thalidomide or lenalidomide) and PIs (bortezomib or carfilzomib) have a median overall survival (OS) of only 13 months [5]. Although patients with relapsed/refractory MM (RRMM) commonly receive triplet/combination therapy [6, 7], novel agents such as daratumumab, a CD38-targeting IgG1Κ human monoclonal antibody, and carfilzomib have demonstrated efficacy as monotherapy in these patients and are approved for such use in the United States [8, 9]. Additional agents with single agent activity and the potential for augmented efficacy in combination regimens are needed to improve outcomes in patients with RRMM.

CD38 is a transmembrane glycoprotein that has multiple functions as both a receptor and an enzyme. It is uniformly, and often highly, expressed on malignant plasma cells, making it an attractive target for the treatment of MM [10, 11]. Isatuximab is an IgG1-kappa monoclonal antibody that binds CD38 via a specific epitope distinct from that of daratumumab [12]. Isatuximab induces tumor cell death via multiple mechanisms, including antibody-dependent cellular cytotoxicity (ADCC), antibody-dependent cellular phagocytosis, complement-dependent cytotoxicity (CDC), and direct apoptosis, and may also activate an immune response against tumor cells through regulation of the tumor immunosuppressive environment via modulation of adenosine levels [12–14].

In a Phase 1 dose escalation study of single agent isatuximab in patients with RRMM who had previously received an IMiD and a PI, the overall response rate (ORR) was 24% (15/63 patients) at doses 10–20 mg/kg, including one complete response (CR) [15]. We report results from part 1 of a Phase 2 study that evaluated the safety and efficacy of isatuximab monotherapy to select the optimal dose and schedule in heavily pretreated patients for use in part 2; results from part 2 evaluating the efficacy of isatuximab alone and in combination with dexamethasone at the dose selected from part 1 will be reported separately.

## Subjects and methods

### Study design

This Phase 2, open-label, randomized, international, multicenter study (NCT01084252) was conducted across 17 study centers (14 in the USA, 2 in Spain, and 1 in Greece). The protocol was approved by institutional review boards/ethics committees and patients provided written informed consent to participate. The study was conducted according to the Declaration of Helsinki and the International Council for Harmonisation of Technical Requirements for Pharmaceuticals for Human Use Good Clinical Practice.

### Study population

Adult patients (≥18 years) with MM were eligible to participate if their disease was refractory to both an IMiD and a PI, or if they had received three or more prior lines of therapy that included an IMiD and PI. All patients must also have received an alkylating agent, have had measurable disease, and obtained a minimal response or better to at least one prior line of therapy, according to International Myeloma Working Group (IMWG) Uniform Response Criteria [16]. Other key inclusion criteria included an Eastern Cooperative Group performance status ≤2 or Karnofsky performance status ≥60 and creatinine clearance ≥30 ml/min. Patients with a stem cell transplant more than 12 weeks prior to starting study treatment were eligible to participate. Patients with IgM myeloma, amyloidosis, myelodysplastic syndrome, or plasma cell leukemia were excluded. Previous anti-CD38 therapy was not permitted, and no other anticancer medications were allowed during the study.

### Treatment

Patients were randomized in a 1:1:1 ratio to one of three dosing regimens, all administered in 28-day cycles. Arm 1 received isatuximab 3 mg/kg every 2 weeks (Q2W), Arm 2 received isatuximab 10 mg/kg Q2W, and Arm 3 received isatuximab 10 mg/kg Q2W for two cycles followed by 10 mg/kg every 4 weeks (Q4W; Day 1 of each cycle only). Randomization was carried out via an Interactive Voice Response System/Interactive Web Response System and was stratified by prior treatment with pomalidomide and/or carfilzomib. As a protocol amendment, based on pharmacokinetic (PK) analysis of Phase 1 data, a fourth arm was added for which patients received isatuximab 20 mg/kg once a week (QW) during the first 28-day cycle and Q2W thereafter. Intrapatient dose escalation (not to exceed 20 mg/kg on the Q2W schedule) was permitted at the discretion of the study sponsor and treating physician for patients with progressive disease if the original dose regimen was tolerated for at least one cycle.

Isatuximab was administered at an initial infusion rate of 175 mg/h, which could be increased in the absence of infusion reactions (IRs) up to a maximum of 400 mg/h. Prophylaxis was given 15–30 min prior to isatuximab infusion to reduce the risk and severity of IRs, consisting of diphenhydramine 25–50 mg IV, methylprednisolone 100 mg IV, ranitidine 50 mg IV (or equivalents for each), and acetaminophen (paracetamol) 650–1000 mg PO.

Patients continued treatment until disease progression, unacceptable toxicity, or another reason for discontinuation. At the end of treatment, those with disease progression were followed every 3 months until death or study cut-off date and those without disease progression were followed monthly until disease progression, initiation of another anticancer therapy, death, or study cut-off date.

### Outcomes

The primary efficacy endpoint was ORR, which included stringent CR, CR, very good partial response (VGPR), or partial response (PR). Responses were assessed by the Independent Adjudication Committee (IAC) using the IMWG Uniform Response Criteria [16], and were determined from central laboratory M-protein (serum and 24-h urine), serum free light chain and serum β2-microglobulin measurements, bone marrow biopsy/aspiration, radiologic imaging of plasmacytoma, and bone skeletal survey. Secondary efficacy endpoints included duration of response, clinical benefit rate, progression-free survival (PFS), and OS. Subgroup analyses were performed for characteristics of interest: age, sex, International Staging System stage, transplant history, high-risk cytogenetics (del[17p] or t[4:14]), renal function, and prior therapies. Patients were termed double refractory if they were refractory to both an IMiD and PI, and quadruple refractory if they were refractory to lenalidomide, pomalidomide, bortezomib, and carfilzomib.

Exploratory endpoints included analysis of Fc gamma receptor polymorphism from baseline blood samples and CD38 receptor density from baseline bone marrow aspirates to evaluate correlations with clinical response.

Disease assessment was on Day 1 of Cycle 2, and Day 1 of every subsequent cycle thereafter. Safety was evaluated based on physical examination, laboratory tests, and adverse event (AE) reporting, which was graded by the National Cancer Institute Common Terminology Criteria for Adverse Events v4.03. High-risk cytogenetics, defined as t(4:14) and/or del(17p), were assessed at a central laboratory using fluorescent in situ hybridization, or local assessment when central data were not available. Blood concentrations of isatuximab were analyzed for population PK using non-linear mixed effects modeling.

### Statistical analyses

A selection design was used to maximize the probability of selecting the best of four isatuximab doses using ORR as the endpoint [17]. Target enrollment was 96 patients (24 patients per arm) to give at least an 80% probability of selecting the best dose assuming an ORR of 10% with 3 mg/kg and a difference in ORR of at least 15% between the best dose and the 3 mg/kg arm.

Analyses of efficacy and safety were based on the all treated population, which comprised all patients who received at least one full or partial administration of isatuximab. For the primary endpoint of ORR, results are summarized as descriptive statistics.

Duration of response, OS, and PFS were analyzed using the Kaplan–Meier method with estimated median and 95% confidence interval (CI). The exploratory analysis of Fc gamma receptor polymorphism used logistic regression analysis. All other analyses are summarized with descriptive statistics.

## Results

### Patients and treatment

The study started on July 2, 2014, with a part 1 data cut-off of December 9, 2016; 12 months after the first dose of the last patient. A total of 97 patients were treated with isatuximab in the dose-finding section of the study.

Patient demographics, disease characteristics, and treatment history are presented by dose group in Table 1. There were no notable imbalances in patient characteristics across the treatment arms. The median age of patients was 62 years (range 38–85) and median time since diagnosis was 5.8 years (range 1.2–24.1). Overall at baseline, 36/97 (37.1%) patients had International Staging System III disease, 33/97 patients (34.0%) had creatinine clearance <60 ml/min, and 28/97 (28.9%) patients had at least one high-risk cytogenetic abnormality (17 with del[17p] and 15 with t[4:14]). The study population had been heavily pretreated, with a median of five prior lines of therapy (range, 2–14). In total, 82/97 (84.5%) patients were double refractory and 32/97 patients (33.0%) were quadruple refractory.

**Table 1:** Patient demographics, disease characteristics, and treatment history (all treated population, *N* = 97).

	Isatuximab dose and schedule				
	3 mg/kg Q2W *n* = 23	10 mg/kg Q2W/Q4W *n* = 25	10 mg/kg Q2W *n* = 24	20 mg/kg QW/Q2W *n* = 25	Total *N* = 97
Age in years, median (range)	63 (44–80)	59 (49–81)	66 (38–83)	59 (48–85)	62 (38–85)
Male, *n* (%)	12 (52.2)	18 (72.0)	13 (54.2)	12 (48.0)	55 (56.7)
Race, *n* (%)					
White	21 (91.3)	19 (76.0)	19 (79.2)	21 (84.0)	80 (82.5)
Black or African American	1 (4.3)	4 (16.0)	2 (8.3)	3 (12.0)	10 (10.3)
Asian	0	0	0	1 (4.0)	1 (1.0)
Other	1 (4.3)	2 (8.3)	3 (12.5)	0	6 (6.2)
Ethnicity, *n* (%)^a^					
Hispanic or Latino	0	1 (4.0)	2 (8.3)	2 (8.0)	5 (5.2)
Not Hispanic or Latino	22 (95.7)	23 (9.0)	22 (91.7)	23 (92.0)	90 (92.8)
ECOG score (Karnofsky PS), *n* (%)					
0 (100%)	7 (30.4)	1 (4.0)	0	8 (32.0)	16 (16.5)
1 (80–90%)	11 (47.8)	22 (88.0)	22 (91.7)	14 (56.0)	69 (71.1)
2 (60–70%)	5 (21.7)	2 (8.0)	2 (8.3)	3 (12.0)	12 (12.4)
Creatinine clearance <60 ml/min, *n* (%)	9 (39.1)	8 (32.0)	10 (41.7)	6 (24.0)	33 (34.0)
Years since initial diagnosis, median (range)	5.8 (2.2–11.0)	5.5 (1.2–12.7)	7.1 (3.4–24.1)	6.1 (1.8–14.3)	5.8 (1.2–24.1)
Measurable paraprotein, *n* (%)^b^					
Serum M-protein	17 (73.9)	20 (80.0)	17 (70.8)	17 (68.0)	71 (73.2)
Urine M-protein	1 (4.3)	4 (16.0)	5 (20.8)	5 (20.0)	15 (15.5)
κ light chain	3 (13.0)	0	1 (4.2)	3 (12.0)	7 (7.2)
λ light chain	1 (4.3)	1 (4.0)	1 (4.2)	0	3 (3.1)
ISS stage at baseline, *n* (%)^b^					
I	5 (21.7)	8 (32.0)	6 (25.0)	11 (44.0)	30 (30.9)
II	8 (34.8)	5 (20.0)	10 (41.7)	7 (28.0)	30 (30.9)
III	9 (39.1)	12 (48.0)	8 (33.3)	7 (28.0)	36 (37.1)
Bone marrow plasma cells, median % (range)	45.0 (1.4–95.0)	24.6 (1.0–97.0)	17.0 (0.0–81.8)	25.5 (1.4–90.0)	24.6 (0.0–97.0)
Extramedullary plasmacytoma at baseline, *n* (%)	4 (17.4)	7 (28.0)	4 (16.7)	2 (8.0)	17 (17.5)
High-risk cytogenetics^c^, *n* (%)	6 (26.1)	5 (20.0)	7 (29.2)	10 (40.0)	28 (28.9)
del(17p)	2 (8.7)	3 (12.0)	5 (20.8)	7 (28.0)	17 (17.5)
t(4:14)	5 (21.7)	2 (8.0)	3 (12.5)	5 (20.0)	15 (15.5)
Median prior lines of therapy, *n* (range)	5 (2–12)	5 (3–14)	6 (2–13)	5 (2–10)	5 (2–14)
≥1 prior stem cell transplant, *n* (%)	20 (87.0)	23 (92.0)	21 (87.5)	22 (88.0)	86 (88.7)
Refractory to an immunomodulatory drug, *n* (%)^d^	21 (91.3)	23 (92.0)	22 (91.7)	23 (92.0)	89 (91.8)
Refractory to lenalidomide, *n* (%)	19 (82.6)	20 (80.0)	20 (83.3)	22 (88.0)	81 (83.5)
Refractory to pomalidomide, *n* (%)	17 (73.9)	16 (64.0)	16 (66.7)	13 (52.0)	62 (63.9)
Refractory to a proteasome inhibitor, *n* (%)^d^	19 (82.6)	23 (92.0)	22 (91.7)	23 (92.0)	87 (89.7)
Refractory to bortezomib, *n* (%)	17 (73.9)	22 (88.0)	16 (66.7)	17 (68.0)	72 (74.2)
Refractory to carfilzomib, *n* (%)	12 (52.2)	17 (68.0)	14 (58.3)	16 (64.0)	59 (60.8)
Refractory to alkylating agent, *n* (%)	15 (65.2)	15 (60.0)	17 (70.8)	14 (56.0)	61 (62.9)
Double refractory, *n* (%)^e^	18 (78.3)	22 (88.0)	20 (83.3)	22 (88.0)	82 (84.5)
Refractory to pomalidomide and carfilzomib	11 (47.8)	14 (56.0)	12 (50.0)	9 (36.0)	46 (47.4)
Quadruple refractory, *n* (%)^f^	9 (39.1)	11 (44.0)	7 (29.2)	5 (20.0)	32 (33.0)

*ECOG* Eastern Cooperative Oncology Group, *ISS* International Staging System, *PS* performance status, *QnW* once every *n* weeks.

^a^Data missing for one patient each in the 3 mg/kg Q2W and 10 mg/kg Q2W/Q4W arms.

^b^Data missing for one patient in the 3 mg/kg Q2W arm.

^c^High-risk cytogenetics defined as t(4:14) translocation and/or 17p deletion. Cytogenetic status unknown for t(4:14) in 19 patients (4 patients in 3 mg/kg Q2W arm, 7 patients in 10 mg/kg Q2W/Q4W, 6 patients in 10 mg/kg Q2W, and 2 patients in 20 mg/kg QW/Q2W arms; and for del(17p) in 19 patients (3 patients in 3 mg/kg Q2W arm, 8 patients in 10 mg/kg Q2W/Q4W, 6 patients in 10 mg/kg Q2W, and 2 patients in 20 mg/kg QW/Q2W arms).

^d^Refractory disease defined according to International Myeloma Working Group criteria.

^e^Double refractory was defined as refractory to an immunomodulatory agent and a proteasome inhibitor.

^f^Quadruple refractory was defined as refractory to lenalidomide, bortezomib, pomalidomide, and carfilzomib.

**Fig. 1:**
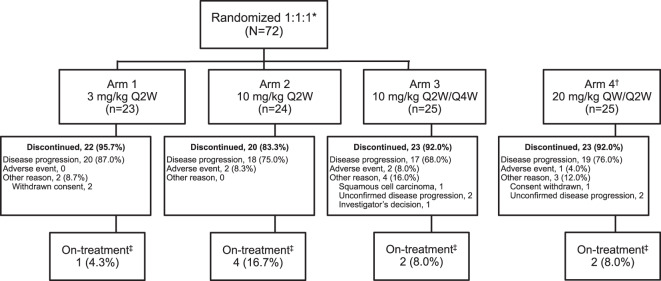
Study design and treatment disposition. QnW every *n* week. *Randomization to Arms 1–3 was stratified according to whether or not patients had received prior treatment with pomalidomide and/or carfilzomib. ^†^Analysis of the pharmacokinetic parameters of patients treated at 10 mg/kg Q2W in the expansion cohort of the Phase 1 study demonstrated a high level of variability in exposure and non-linear clearance, suggesting that a higher dose and more intense “loading” schedule may be required to reach the desired therapeutic concentration faster. Therefore, Arm 4 was included, which evaluated a dose and schedule of 20 mg/kg QW for 1 cycle followed by 20 mg/kg Q2W. ^‡^At study cut-off, December 9, 2016.

Patient disposition is shown in Fig. 1. In the overall population, patients received a median 3 cycles (range 1–19) and the median duration of treatment was 13 weeks (range 2–77). In the dose groups ≥10 mg/kg, the median number of cycles was 4 and median duration of exposure was between 14.0 and 15.9 weeks across the three cohorts. The median duration of isatuximab infusion was reduced between the first infusion and subsequent infusions, from 3.2 to 2.6 h for 10 mg/kg, and 5.3 to 4.4 h for 20 mg/kg.

### Efficacy

The ORR by IAC assessment for all dose groups was 19.6% (19/97 patients), including 11.3% (11/97 patients) with a VGPR and 8.2% (8/97) with a PR; no CR were observed. The ORR based on investigator assessment was similar at 21.6% (21/97 patients). The ORR by dose (Supplementary Fig. 1a) was 4.3% in the isatuximab 3 mg/kg arm arising from one PR, 20.0% (5/25 patients) at 10 mg/kg Q2W/Q4W, 29.2% (7/24) at 10 mg/kg Q2W, and 24.0% (6/25) at 20 mg/kg QW/Q2W. At doses ≥10 mg/kg, the ORR was 24.3% (18/74 patients), which included 14.9% (11/74) of patients with a VGPR and 9.5% (7/74) with a PR. The overall clinical benefit rate was 28.9% (28/97 patients) by IAC and 30.9% (30/97 patients) by investigator assessment. By dose group, the clinical benefit rate was 4.3% (1/23 patients) for 3 mg/kg Q2W, 41.7% (10/24) for 10 mg/kg Q2W, 32.0% (8/25) for 10 mg/kg Q2W/Q4W, and 36.0% (9/25) for 20 mg/kg QW/Q2W.

The ORR at doses ≥10 mg/kg was maintained in subgroup analyses of patients with baseline characteristics indicative of a poor prognosis (Supplementary Fig. 1b). Notably, at doses ≥10 mg/kg, the ORR was 46.2% (6/13) vs 19.7% (12/61) in patients aged ≥70 years vs <70 years, and 40.9% (9/22) vs 17.3% (9/52) in patients with vs without high-risk cytogenetic markers, respectively. By cytogenetic abnormality, the ORR was 40.0% (6/15 patients) for del(17p) and 40.0% (4/10 patients) for t(4:14). Furthermore, at doses ≥10 mg/kg, the ORR varied little by prior treatment history, with 23.3% (14/60) of patients with three or more previous lines of therapy, 25.0% (16/64) of patients with double refractory disease, and 17.4% (4/23) of patients with quadruple refractory disease achieving at least a PR.

Patients responded early to treatment and these responses were durable (Supplementary Fig. 2). Median time to first response was 1.8 months (range 0.9–5.7). The duration of response was 1.9 months in the one responder in the 3 mg/kg arm, 8.3 months (3.7–12.4) in the 10 mg/kg Q2W/Q4W group, 14.8 months (3.7–16.6) in the 10 mg/kg Q2W group, and 8.3 months (3.7–10.2) in the 20 mg/kg QW/Q2W group.

**Fig. 2:**
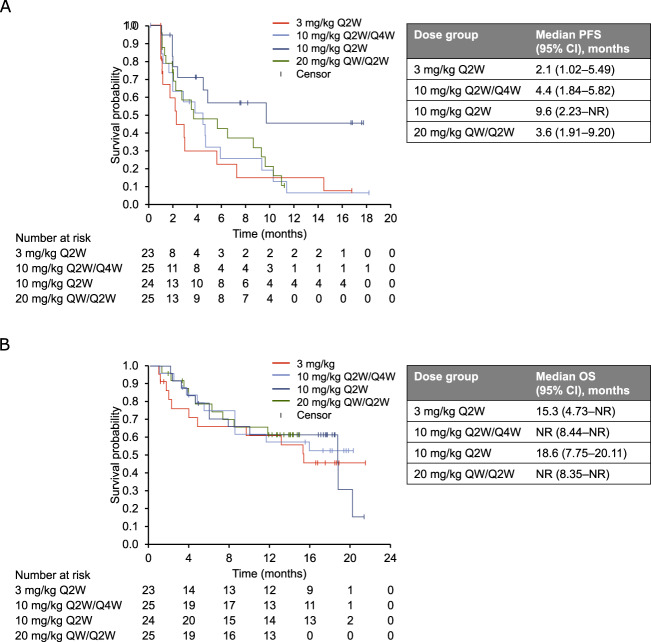
Survival (all treated population; *N* = 97). **a** Progression-free survival. **b** Overall survival.

Median PFS ranged from 2.1 months in the 3 mg/kg Q2W arm to 9.6 months in the 10 mg/kg Q2W arm (Fig. 2a). Median OS was 15.3 months in the 3 mg/kg Q2W group, 18.6 months in the 10 mg/kg Q2W group, and not reached in the 10 mg/kg Q2W/Q4W and 20 mg/kg QW/Q2W groups (Fig. 2b). At doses ≥10 mg/kg, median PFS was 4.6 months (95% CI, 2.8–9.2) and median OS was 18.7 months (95% CI, 11.7–not reached).

### Safety

Almost all patients had at least one treatment-emergent AE (TEAE; 96/97 [99.0%] patients) and 66/97 (68.0%) patients had a grade ≥3 TEAE. The most common any grade TEAEs (≥20% of patients), grade ≥3 TEAEs (≥5% of patients), and hematologic abnormalities are presented in Table 2. IRs were the most commonly reported non-hematologic TEAE, reported in 50/97 (51.5%) patients, and were also the most frequently reported drug-related TEAE. IRs mostly occurred with the first infusion, with 5/97 (5.2%) patients experiencing IRs during the second infusion or later, and were mostly grade 1 or 2 in severity, with only two cases of grade ≥3 IRs. There was no difference in IR incidence between 10 and 20 mg/kg dose levels (56.0–58.3 vs 56.0%), however, incidence was lower at 3 mg/kg (34.8%).

**Table 2:** Most common TEAEs (≥20% patients), grade ≥3 TEAEs (≥5% patients), and hematologic abnormalities (all treated population; *N* = 97).

	Isatuximab dose and schedule									
	3 mg/kg Q2W (*n* = 23)		10 mg/kg Q2W/Q4W (*n* = 25)		10 mg/kg Q2W (*n* = 24)		20 mg/kg QW/Q2W (*n* = 25)		Total (*N* = 97)	
	All grades	Grade 3/4	All grades	Grade 3/4	All grades	Grade 3/4	All grades	Grade 3/4	All grades	Grade 3/4
TEAEs, *n* (%)	22 (95.7)	17 (73.9)	25 (100)	15 (60.0)	24 (100)	19 (79.2)	25 (100)	15 (60.0)	96 (99.0)	66 (68.0)
Nausea	6 (26.1)	0	11 (44.0)	0	9 (37.5)	0	7 (28.0)	0	33 (34.0)	0
Fatigue	5 (21.7)	0	11 (44.0)	0	6 (25.0)	0	9 (36.0)	0	31 (32.0)	0
URTI	6 (26.1)	2 (8.7)	9 (36.0)	0	7 (29.2)	0	6 (24.0)	1 (4.0)	28 (28.9)	3 (3.1)
Diarrhea	5 (21.7)	0	9 (36.0)	1 (4.0)	7 (29.2)	0	5 (20.0)	1 (4.0)	26 (26.8)	2 (2.1)
Cough	2 (8.7%)	0	7 (28.0)	0	9 (37.5)	0	8 (32.0)	0	26 (26.8)	0
Headache	4 (17.4)	0	8 (32.0)	0	4 (16.7)	0	7 (28.0)	0	23 (23.7)	0
Dyspnea	5 (21.7)	0	8 (32.0)	1 (4.0)	5 (20.8)	1 (4.2)	4 (16.0)	0	22 (22.7)	2 (2.1)
Pneumonia	2 (8.7)	1 (4.3)	2 (8.0)	2 (8.0)	4 (16.7)	4 (16.7)	0	0	8 (8.2)	7 (7.2)
Progressive disease	4 (17.4)	4 (17.4)	1 (4.0)	1 (4.0)	0	0	1 (4.0)	1 (4.0)	6 (6.2)	6 (6.2)
Sepsis	4 (17.4)	4 (17.4)	0	0	1 (4.2)	1 (4.2)	0	0	5 (5.2)	5 (5.2)
Hematologic abnormalities, *n*/*n* (%)^a^										
Anemia	22/22 (100)	7/22 (31.8)	23/24 (95.8)	9/24 (37.5)	21/22 (95.5)	4/22 (18.2)	25/25 (100)	3/25 (12.0)	91/93 (97.8)	23/93 (24.7)
White blood cell decreased	14/22 (63.6)	2/22 (9.1)	18/24 (75.0)	0	15/22 (68.2)	5/22 (22.7)	24/25 (96.0)	4/25 (16.0)	71/93 (76.3)	11/93 (11.8)
Lymphocyte count decreased	12/22 (54.5)	5/22 (22.7)	17/24 (70.8)	7/24 (29.2)	19/22 (86.4)	7/22 (31.8)	21/25 (84.0)	8/25 (32.0)	69/93 (74.2)	27/93 (29.0)
Platelet count decreased	14/22 (63.6)	5/22 (22.7)	14/24 (58.3)	2/24 (8.3)	11/22 (50.0)	2/22 (9.1)	20/25 (80.0)	6/25 (24.0)	59/93 (63.4)	15/93 (16.1)
Neutrophil count decreased	8/22 (36.4)	5/22 (22.7)	7/24 (29.2)	1/24 (4.2)	8/22 (36.4)	6/22 (27.3)	15/25 (60.0)	6/25 (24.0)	38/93 (40.9)	18/93 (19.4)
Adverse events of special interest										
Infusion reaction	8 (34.8)	0	14 (56.0)	0	14 (58.3)	2 (8.3)	14 (56.0)	0	50 (51.5)	2 (2.1)

*TEAE* treatment-emergent adverse event, *QnW* once every *n* weeks, *URTI* upper respiratory tract infection.

^a^Based on clinical laboratory data.

Besides IRs, drug-related TEAEs reported in ≥5% of patients consisted of chills (16.5%), nausea (15.5%), dyspnea (12.4%), chest discomfort (11.3%), flushing (11.3%), cough (8.2%), headache (7.2%), vomiting (6.2%), and wheezing (5.2%). Except for dyspnea, which occurred at grade ≥3 in 1 patient (1.0%), none of these drug-related TEAEs were reported at grade >2.

The most frequently reported grade ≥3 TEAEs were hematologic abnormalities and pneumonia (Table 2). Grade 3 febrile neutropenia occurred in one patient in the 3 mg/kg Q2W arm, but no neutropenic infections were observed. Four patients (4.1%) had a serious drug-related TEAE, comprising one anaphylactic reaction with bronchospasm and one varicella zoster infection in the 10 mg/kg Q2W group, and one case of gastroenteritis and one case of meningococcal sepsis in the 20 mg/kg QW/Q2W group.

Five AEs (5.2%) led to withdrawal from the study, of which two were IRs: one grade 4 IR (anaphylactic reaction and bronchospasm), one grade 3 IR, one sudden death (not considered related to isatuximab), one grade 3 platelet count decrease, and one grade 5 atrial fibrillation (not considered related to isatuximab). There were eight (8.2%) deaths during the treatment period: three due to AEs (cerebral hemorrhage, atrial fibrillation, and sudden death), and five due to progressive disease, none of which were considered related to treatment.

None of the 94 evaluable patients were positive for antidrug antibodies at baseline or after isatuximab administration.

### Exploratory analyses: CD38 receptor density and Fc gamma receptor polymorphism

Of the 60 patients evaluable for CD38 receptor density at baseline, median CD38 receptor density was 151,317 molecules/cell (range 53,307–539,799) in 15 responders and 143,408 molecules/cell (49,354–349,722) in 45 non-responders. At active doses ≥10 mg/kg, the ORR for patients with CD38 receptor density above or below the threshold value of 150,000 molecules/cell was 33.3% (8/24 patients) and 27.3% (6/22 patients), respectively.

The prevalence of FcγRIIIA (CD16) cytogenetic variants was 10.7% (10/93 patients) FcγRIIIA V/V, 39.8% (37/93) F/F, and 49.5% (46/93) F/V. The highest ORR (60.0%; 6/10 patients) was observed in the V/V variant population, compared with 8.1% (3/37) for F/F and 19.6% (9/46) for F/V.

### PK

Based on the geometric means ratio, increasing the dose of isatuximab 6.7-fold from 3 to 20 mg/kg resulted in a dose-proportional increase in the maximum concentration (*C*_max_) at Cycle 1 Day 1 (6.9-fold increase), whereas area under the curve (AUC) at Week 1 (AUC 1W) increased in a greater than dose-proportional manner (8.5-fold increase). When the dose was increased 2-fold, from 10 to 20 mg/kg, there was a dose-proportional increase in *C*_max_ and AUC 1W (both 1.8-fold increase) (Table 3). This suggests the presence of target-mediated drug disposition with isatuximab. For the 10 mg/kg Q2W schedule, the accumulation ratio for *C*_max_ was 1.3 at Cycle 2 and 1.9 at Cycle 6. For the 10 mg/kg Q2W/Q4W schedule, an accumulation in *C*_max_ and *C*_trough_ was observed at Cycle 2, but no longer at Cycle 6 (*C*_trough_ accumulation ratio, 0.68). For the 20 mg/kg QW/Q2W schedule, accumulation ratios at Cycle 2 were 2.2 for *C*_max_ and 3.3 for *C*_trough_, and were similar at Cycle 6 (2.3 and 3.6, respectively), indicating that steady state is reached by Cycle 2 for this administration schedule (Table 4).

**Table 3:** Geometric means for isatuximab AUC 1W and *C*_max_ across the dose range investigated.

Isatuximab dose and regimen	AUC 1W (µg.h/ml)	*C*_max_ C1D1 (µg/ml)
3 mg/kg Q2W (*n* = 23)	3023	41
10 mg/kg Q2W (*n* = 21)	13,837	153
10 mg/kg Q2W/Q4W (*n* = 25)	13,713	154
10 mg/kg Q2W for C1^a^ (*n* = 46)	13,769	154
20 mg/kg QW/Q2W (*n* = 24)	25,596	279

*AUC 1W* predicted cumulative area under the plasma concentration curve over the first week (0–168 h), *C* cycle, *C1D1* cycle 1, day 1, *C*_*max*_ maximum plasma concentration, *QnW* every *n* weeks.

^a^C1–C2 for *C*_max_.

**Table 4:** Exposure (*C*_max_, *C*_trough_, and AUC_0−τ_) parameters for isatuximab in a typical patient, with accumulation ratios.

	Cycle 1			Cycle 2		Steady state		
	*C*_max_ (µg/ml)	*C*_trough_ (µg/ml)	AUC_0−τ_ (µg.h/ml)	*C*_max_ (µg/ml)	*C*_trough_ (µg/ml)	*C*_max_ (µg/ml)	*C*_trough_ (µg/ml)	AUC_0−τ_ (µg.h/ml)
10 mg/kg Q2W	142	24.7	18,288	190	66.7	272	132	58,557
Accumulation ratio				1.34	2.70	1.92	5.35	3.20
10 mg/kg Q2W/Q4W	142	24.7	18,288	190	66.7	163.4	16.8	36,090
Accumulation ratio				1.34	2.70	1.15	0.68	–
20 mg/kg QW/Q2W	278	103	26,711	617	335	651	369	152,884
Accumulation ratio				2.22	3.26	2.34	3.60	–

*AUC*_*0−τ*_ predicted cumulative area under the plasma concentration curve from time zero to time τ, *C*_*max*_ maximum plasma concentration, *C*_*trough*_ plasma concentration before treatment administration during repeated dosing, *QnW* every *n* week.

## Discussion

In this study in heavily pretreated patients with RRMM, single agent isatuximab showed promising efficacy at doses ≥10 mg/kg, with 24.3% of patients demonstrating a response to treatment and 14.9% achieving a VGPR, with a median PFS of 4.6 months and median OS of 18.7 months. These responses were durable, lasting from 8.3 to 14.8 months depending on dose schedule, and occurred early, often at the first disease assessment (median time to first response, 1.8 months).

The efficacy of isatuximab is important given the prior treatment burden of patients in this study, comprising a median five previous lines of therapy and 85% dual refractoriness to IMiDs and PIs. It is well recognized that responses diminish with successive treatment regimens [3], and real-world studies in similar patient populations highlight the poor prognosis (median OS of 8–13 months) of these patients [5, 18].

Responses to isatuximab were consistently demonstrated in all high-risk groups analyzed, including those with more than three prior lines of therapy, double and quadruple refractory disease, ≥70 years of age, and at least one high-risk cytogenetic abnormality. Indeed, the ORR was higher in older and high-risk cytogenetic patients than the overall population, although small subgroup numbers warrant caution when interpreting the data. The efficacy of isatuximab in these hard-to-treat patients is promising, especially given real-world data showing reduced response rates and OS in older patients compared with their younger counterparts [19–21] and reduced median OS in patients with high-risk cytogenetics vs standard-risk patients [22, 23].

The results of this study with isatuximab monotherapy compare favorably with those from studies supporting approved single agents in RRMM. In the Phase 2 SIRIUS study of daratumumab, patients had received an alkylating agent and at least three prior lines of therapy, including a PI and IMiD, or were double refractory to the last PI and IMiD received. In the daratumumab 16 mg/kg arm (*n* = 106), patients had a median five prior lines of therapy (range 2–14) and 95% were double refractory to a PI and IMiD. The ORR (primary endpoint) was 29.2% (including 3 CRs and 10 VGPRs, corresponding to 12.3% of patients achieving ≥VGPR), median PFS was 3.7 months and median OS was 17.5 months [24]. In a Phase 2 study of carfilzomib in 266 patients with MM who had received at least two prior therapies, including bortezomib and an IMiD, the ORR (primary endpoint) was 23.7%, median OS was 15.4 months, and median PFS was 3.7 months [25, 26]. A Phase 3 study investigated single agent carfilzomib vs low-dose corticosteroid and optional cyclophosphamide in patients who had received at least three prior treatments, including bortezomib, lenalidomide, or thalidomide, an alkylating agent and corticosteroids, and who were refractory to their most recent therapy. The primary endpoint of median OS was 10.2 months in the carfilzomib group compared with 10.0 months in the control group, and median PFS was 3.7 vs 3.3 months, respectively [27].

Isatuximab was generally well tolerated at doses ≥10 mg/kg, with no observed dose-dependency in terms of type, incidence, and severity of TEAEs. Few patients discontinued treatment due to AEs. With the administration of prophylactic premedications, and no mandated postinfusion corticosteroids, IRs occurred in approximately half of patients, mostly during the first infusion, and were mild-to-moderate in almost all cases. The duration of isatuximab infusion could be reduced after the first infusion (to 2.6 h for 10 mg/kg and 4.4 h for 20 mg/kg), without incurring any increased risk of IRs.

A previous study found an association between receptor density and response to daratumumab, reflecting an observed link between CD38 expression levels on patient MM cells and induced cell death by daratumumab via ADCC and CDC [28, 29]. In this study, an exploratory analysis of CD38 receptor density did not detect any association with clinical response to isatuximab. Although limited by the number of patients evaluable, these preliminary findings would caution against the use of CD38 expression levels as a predictive marker of isatuximab efficacy. Further exploration with other next-generation CD38 monoclonal antibodies such as SAR442085 is recommended.

Isatuximab induces both Fc-dependent and Fc-independent pathways to kill CD38-expressing tumor cells [12, 13]. Of these, Fc-dependent natural killer cell-mediated ADCC is a key mechanism that occurs in both low and high CD38-expressing tumor plasma cells [14]. The affinity of the natural killer cell FcγRIIIA receptor to the Fc portion of IgG antibodies is increased by a naturally occurring F to V mutation [30]. Exploratory analyses in this study revealed a higher ORR in patients with the high-affinity FcγRIIIA V/V variant than patients with the lower-affinity F/F or F/V variants. Larger studies are needed to determine whether these findings are generalizable in a broader MM population and whether FcγRIIIA variant status could be predictive of response to isatuximab, including in combination therapy.

This study confirms the findings from a Phase 1 study of single agent isatuximab in heavily pretreated patients with RRMM (median 5 prior lines of therapy, including an IMiD and PI), where the ORR was 24% at doses ≥10 mg/kg [15]. There was no clear dose–response relationship between 10 and 20 mg/kg. PK/pharmacodynamic modeling and simulations of ORR and serum M-protein (a surrogate for tumor growth) showed better efficacy with a deeper response in terms of serum M-protein reduction at dose 20 mg/kg compared with 10 mg/kg, and 20 mg/kg was subsequently chosen as the optimal dose for single agent isatuximab [31]. This decision is supported by PK data from this study; steady state was reached by Cycle 2 with the 20 mg/kg QW/Q2W regimen, whereas variations in accumulation ratios for *C*_max_ and *C*_trough_ between Cycle 2 and Cycle 6 were observed with both 10 mg/kg regimens. Furthermore, PK data from a Phase 1 study of isatuximab in patients with RRMM or other CD38-positive hematologic malignancies indicate that, at 10 mg/kg Q2W, Cycle 1 plasma trough levels of isatuximab are consistently above the lowest pharmacological active dose in mouse tumor models (10 μg/ml), and at dose 20 mg/kg Q2W, Cycle 2 trough levels are above the concentration needed for tumor eradication (129 μg/ml) [32]. Notably, PFS appears to drop after 4 months in the 10 mg/kg Q2W/Q4W cohort compared with the other highest dosing groups, suggesting that Q2W administration after loading is important to maintain a response in patients with advanced MM.

As patients were not randomized to the 20 mg/kg QW/Q2W group, direct comparisons cannot be made between this and the other study arms. It is also noteworthy that detection of the therapeutic monoclonal antibody may obscure whether a patient with an IgG-kappa M-spike has attained a complete hematologic remission. A CR per IMWG criteria requires the absence of M-protein by immunofixation, while a VGPR allows M-protein detectable by immunofixation but not on electrophoresis [16, 33]. Higher interference from isatuximab at the 20 mg/kg dose may contribute to a lower VGPR rate at this dose level compared with lower doses. At the time the study was conducted, there was no available assay to detect, and therefore remove, this interference.

Part 2 of this study is ongoing to evaluate isatuximab 20 mg/kg as monotherapy or in combination with dexamethasone in patients with RRMM (NCT01084252). Isatuximab is also being investigated in combination regimens [34–36] and Phase 3 trials are underway; the ICARIA study has shown that addition of isatuximab to pomalidomide-dexamethasone improves the ORR and PFS, including in patients with adverse characteristics such as high-risk cytogenetics, heavy pretreatment, and renal impairment (NCT02990338) [35, 37–40]. Other Phase 3 trials are evaluating the addition of isatuximab to lenalidomide, bortezomib, and dexamethasone (RVd) in patients with newly diagnosed MM (NCT03617731 and NCT03319667), and carfilzomib and dexamethasone in patients with RRMM (NCT03275285).

In conclusion, isatuximab demonstrated single agent activity in heavily pretreated patients with RRMM, including high-risk patients, and these responses were more durable at doses ≥10 mg/kg. Isatuximab was generally well tolerated. The time burden of infusions could be safely reduced following the first infusion and most IRs were mild-to-moderate in severity and rarely occurred after the first infusion. Isatuximab is a significant new treatment option for patients with RRMM and studies are ongoing for use both as a single agent and in combination with other treatment regimens.

## Supplementary information

Supplemental Figure legends

Supplemental Figure 1

Supplemental Figure 2
