# Supplementary material for: A dose-finding Phase 2 study of single agent isatuximab (anti-CD38 mAb) in relapsed/refractory multiple myeloma
Source: Leukemia. 2020 May 14;34(12):3298–309. doi: 10.1038/s41375-020-0857-2 (PMC7685976; doi:10.1038/s41375-020-0857-2)
Supplement: Supplementary file 1 — Supplemental Figure legends [file 41375_2020_857_MOESM1_ESM.docx]

Supplemental Figure 1. Overall response rate per IMWG criteria^*^ by dose group (A) and by subgroup^†^ at doses ≥10 mg/kg (B) (all treated population; N=97)

Btz, bortezomib; Car, carfilzomib; CrCl, creatinine clearance; IMWG, International Myeloma Working Group; ; ISS, International Staging System; Len, lenalidomide; ORR, overall response rate; Pom, pomalidomide; PR, partial response; QnW, every n weeks; VGPR, very good partial response

*Confirmed responses, defined according to IMWG criteria for all treated patients. ^†^Data shown for subgroups of more than 10 patients. ^‡^High-risk cytogenetics defined as t(4:14) and/or del(17p).

Supplemental Figure 2. Time on treatment by best response (all treated population; N=97)

PR, partial response; QnW, every n weeks; VGPR, very good partial response

First disease assessment taken at Cycle 2. No complete responses were observed.
